# Foundation models in molecular biology

**DOI:** 10.52601/bpr.2024.240006

**Published:** 2024-06-30

**Authors:** Yunda Si, Jiawei Zou, Yicheng Gao, Guohui Chuai, Qi Liu, Luonan Chen

**Affiliations:** 1 Key Laboratory of Systems Health Science of Zhejiang Province, School of Life Science, Hangzhou Institute for Advanced Study, University of Chinese Academy of Sciences, Chinese Academy of Sciences, Hangzhou 310024, China; 2 Shanghai Institute of Biochemistry and Cell Biology, Center for Excellence in Molecular Cell Science, Chinese Academy of Sciences, University of Chinese Academy of Sciences, Shanghai 200031, China; 3 Translational Medical Center for Stem Cell Therapy and Institute for Regenerative Medicine, Shanghai East Hospital, Frontier Science Center for Stem Cell Research, Bioinformatics Department, School of Life Sciences and Technology, Tongji University, Shanghai 200092, China; 4 Shanghai Research Institute for Intelligent Autonomous Systems, Shanghai 201804, China

**Keywords:** Foundation models, Molecular biology, Transcriptome

## Abstract

Determining correlations between molecules at various levels is an important topic in molecular biology. Large language models have demonstrated a remarkable ability to capture correlations from large amounts of data in the field of natural language processing as well as image generation, and correlations captured from data using large language models can also be applicable to solving a wide range of specific tasks, hence large language models are also referred to as foundation models. The massive amount of data that exists in the field of molecular biology provides an excellent basis for the development of foundation models, and the recent emergence of foundation models in the field of molecular biology has really pushed the entire field forward. We summarize the foundation models developed based on RNA sequence data, DNA sequence data, protein sequence data, single-cell transcriptome data, and spatial transcriptome data respectively, and further discuss the research directions for the development of foundation models in molecular biology.

## INTRODUCTION

**Figure 1:**
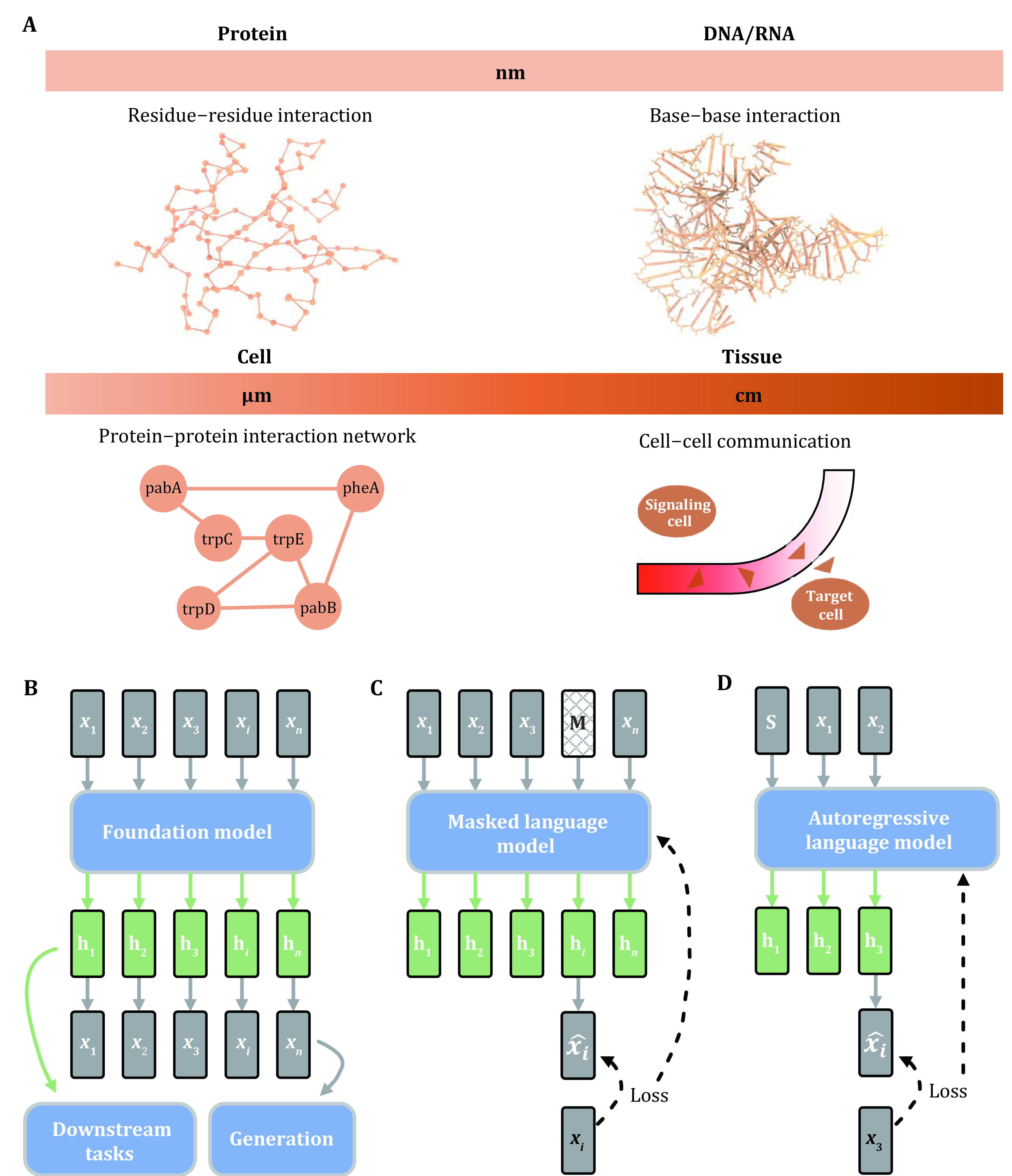
Overview of molecular interactions and foundation models. **A** Types of molecular interactions at different spatial scales. **B** Processes for representing data as embeddings using foundation models and using the embeddings for downstream tasks. Where x*i* denotes the *i*^th^ element in the data X, h*i* denotes the embedding corresponding to the *i*th element. **C** Masked language model learns the correlation between elements in the data by masking a portion of the elements in the data (denoted as M) and then using the remaining portion to predict the masked elements, the difference between the predicted value of the masked portion and the true value is used to update the model. **D** Autoregressive language model learns the correlation between elements in the data by sequentially predicting the next element in the data from the beginning (denoted as S), and the difference between the predicted and true value of the next element is used to update the model

Interactions between biomolecules (such as metals, proteins, lipids, and nucleic acids) involve in numerous life processes at different spatial scales (Fig. 1A), which are essential for the maintenance of normal life activities (Limo *et al*. 2018; Nooren and Thornton 2003; Tiwari and Chakrabarty 2021; Jankowsky and Harris 2015). For example, interactions between residues determine the folding path of proteins and the structure formed by folding, and misfolding can lead to abnormal protein function (Dobson 1999; Hartl 2017); interactions between proteins are essential for intercellular signaling and intracellular catalysis (Henderson and Pockley 2010; Zheng *et al*. 2023). Decoding the interaction networks of biomolecules is a central challenge in the field of molecular biology, and a comprehensive understanding of the interaction network of biomolecules will not only dramatically advance the understanding of life processes and the treatment of diseases, but will also enable the construction of numerical models of biological systems that are capable of precise biomolecular experimentation.

There are various types of interactions between biomolecules, such as protein–protein interactions, RNA-small molecule interactions, *etc*., and numerous approaches have been used to characterize the interactions between biomolecules (Gao *et al*. 2023; Lenz *et al*. 2021; Mann *et al*. 2017; Sledzieski *et al*. 2021; Umu and Gardner 2017). Biological experiments are commonly used to characterize the interactions between biomolecules (Bai *et al*. 2015; Nguyen *et al*. 2016; Rual *et al*. 2005), and the combination of high-throughput and low-throughput experiments has generated a lot of valuable data, for example, 3.7 million pairs of RNA–RNA interactions discovered by experiments have been stored in the starBase (Li *et al*. 2014) database, and BioGRID (Oughtred *et al*. 2021) database has also stored 2.7 million pairs of protein-protein interactions discovered by experiments. However, it is estimated that experimentally discovered interactions between biomolecules still represent only a small fraction of all possible interactions (Lu *et al*. 2020; Ramanathan *et al*. 2019). Computational approaches are important complements to experimental approaches in determining whether interactions exist between biomolecules, and tend to have a significant advantage in speed over experimental approaches, while accuracy may have some limitations (Cirillo *et al*. 2012; McDowall *et al*. 2009; Puton *et al*. 2012; Rao *et al*. 2014). Deep learning approaches are the significant breakthrough in computational approaches, which are good at learning interaction patterns from existing interactions between biomolecules and then applying such learned knowledge to explore undiscovered interactions between biomolecules (Gao *et al*. 2023; Li *et al*. 2022b; Singh *et al*. 2022). The performance of deep learning approaches has been greatly improved compared to traditional approaches, but the lack of interaction data has also limited the performance of deep learning approaches.

In contrast to the seriously scarce task-specific data, the huge amount of unlabeled data is another yet-to-be-explored treasure within the field of molecular biology (including protein sequences, DNA sequences, RNA sequences, single-cell transcriptome data, *etc*., see Table 1), for example, there are only about 500,000 experimental protein structures (as determined by residue–residue interactions) in the Protein Data Bank (Goodsell *et al*. 2020), whereas the number of protein sequences contained in the BFD protein sequence database is already 2.5 billion (Jumper *et al*. 2021). These unlabeled data are “snapshots” of the interactions between biomolecules, protein sequences reveal which residues are arranged in which order to fold into a stable protein structure, while single-cell transcriptome data imply the regulatory relationships between genes. How to distill the correlations between biomolecules from these unlabeled data is another important question, and this is an area in which language models can specialize. Language modeling has been very widely used in molecular biology after its great success in the field of natural language processing and has led to the research paradigm of “pre-training + fine-tuning” (Bepler and Berger 2021; Devlin *et al*. 2019; Dodge *et al*. 2020; Vaswani *et al*. 2017; Wang *et al*. 2023e). In this review, we first briefly describe the architectures of common language models, then report the performance and application scenarios of language models developed based on RNA sequence data, protein sequence and structure data, and single-molecule transcriptome data, and finally discuss the next steps in the development of language models in molecular biology.

**Table 1:** Foundation models in molecular biology

Type	Name	Network	Input	Parameters	Pre-training database
Protein	SeqVec (Heinzinger *et al*. 2019)	ELMo	Sequence	93.6M	Uniref50
	TAPE ( Rao *et al*. 2019)	LSTM	Sequence	N/A	Pfam
		ResNet		N/A	
		Transformer		38M	
	ESM-1b (Rives *et al*. 2021)	BERT	Sequence	650M	Uniref50
	ProtT5 (Elnaggar *et al*. 2022)	T5	Sequence	11B	Uniref50
	ProtTXL (Elnaggar *et al*. 2022)	Transformer-XL	Sequence	562M	BFD, Uniref100
	OmegaPLM (Wu *et al*. 2022)	BERT	Sequence	670M	Uniref50
	ESM-2 (Lin *et al*. 2023)	BERT	Sequence	15B	Uniref50
	MSA-transformer (Rao *et al*. 2021)	Axial attention	Multiple sequence alignment	100M	Uniref50, Uniclust30
	ProtGPT2 (Ferruz*et al*. 2022)	GPT2	Sequence	738M	Uniref50
	ProtGen (Madani *et al*. 2023)	Transformer	Sequence, function	1.2B	Uniparc, TrEMBL, *etc*.
DNA	DNABERT (Ji *et al*. 2021)	BERT	Sequence	86M	Human genome
	DNABERT-2 ( Zhou *et al*. 2023b)	BERT	Sequence	117M	135 genomes
	Nucleotide transformer (Dalla-Torre *et al*. 2023)	Transformer encoder	Seuqence	2.5B	850 genomes
	GPN (Benegas *et al*. 2023)	ResNet	Sequence	66M	8 genomes
	DeepCRISPR (Chuai *et al*. 2018)	CNN-based	Sequence	85M	13 genomes and 52 epi-genetic genomes
RNA	RNA-FM (Chen *et al*. 2022)	BERT	Sequence	100M	RNAcentral
	Uni-RNA (Wang *et al*. 2023e)	BERT	Sequence	400M	Nt, RNAcentral, *etc*.
	RNA-MSM (Zhang *et al*. 2023)	Axial attention	Multiple sequence alignment	83M	Rfam
Single celltranscriptome	scBERT (Yang *et al*. 2022)	Performer	Transcriptome	8M	Panglao
	scFormer (Cui *et al*. 2022)	Transformer	Transcriptome	N/A	Cortex, Spleen, *etc*.
	Geneformer (Theodoris *et al*. 2023)	BERT	Transcriptome	52M	Genecorpus-30M
	scGPT (Cui *et al*. 2023)	GPT	Transcriptome	51M	Human cell-33M
	scTranslator (Liu *et al*. 2023)	GPT	Transcriptome	N/A	Bulk data, single cell data, *etc*.

## LANGUAGE MODELS

Understanding words or phrases in their context is a critical challenge in natural language processing, which has been greatly facilitated by the introduction of deep learning, especially large language models. Large language models usually adopt Long Short-Term Memory (LSTM.Pdf n.d.) (LSTM) or Transformer (Vaswani *et al*. 2017) as the backbone network, which is trained with self-supervised learning on a large amount of unlabeled text. The central concept of self-supervised learning is to use the data itself to generate labels and there are two common approaches of self-supervised learning in use today, one is to randomly mask a portion of the text and then use the unmasked portion to predict the content of the masked portion (Devlin *et al*. 2019; He *et al*. 2020; Joshi *et al*. 2018), and the other is to predict what the next word or phrase will be from the previous text (Brown *et al*. 2020; Radford *et al*. 2018, 2019). If the model has the ability to predict the content of the masked portion or what the next word will be from the existing text, then it means that the model does capture the correlations between words and to some extent can understand the meaning of a word in their context. BERT (Devlin *et al*. 2019), ESM-1b (Rives *et al*. 2021) and other works (Brown *et al*. 2020; Cui *et al*. 2020; Dong *et al*. 2019; Radford *et al*. 2018) have proved that large language models do have certain ability to predict the content of the masked portion in the text, while the embedding of words extracted from large language models has been found to contain the contextual context of the corresponding word in the work of Peters *et al*. (Peters *et al*. 2018). Language models trained with the objective of recovering the content of the masked region are superior in text comprehension, and language models trained with the objective of predicting the next word excel in text generation (Ethayarajh 2019; Klein and Nabi 2019). While the performance of language models for different network architectures tends to have some differences, the BERT (Devlin *et al*. 2019) and GPT (Radford *et al*. 2018, 2019) architectures are currently the most widely used language model architectures for their excellent performance in the tasks of understanding text and generating new text. First, we first introduce Transformer, and then describe the architecture and training approaches of BERT and GPT.

### Transformer architecture

Transformer (Vaswani *et al*. 2017) uses an encoder-decoder architecture and achieves excellent performance on machine translation tasks. Where the encoder is used to convert the input sequence into a continuous representation, the decoder uses the output of the encoder as a condition to sequentially predict the words in the translated sentence. Each layer in the encoder and decoder consists of a multi-head attention module and a feed-forward module, the “Scaled Dot-Product Attention” in the multi-head attention module ensures that the encoder considers the entire input when processing each element, and the following is the formula of “Scaled Dot-Product Attention”:

$ \begin{array}{c}Q\\ K\\ V\end{array}=\;\mathrm{X}\;\times\; \left[\begin{array}{c}{W}^{\,Q}\\ {W}^{\,K}\\ {W}^{\,V}\end{array}\right] , $

$ \mathrm{A}\mathrm{t}\mathrm{t}\mathrm{e}\mathrm{n}\mathrm{t}\mathrm{i}\mathrm{o}\mathrm{n}\left(Q,K,V\right)=\mathrm{S}\mathrm{o}\mathrm{f}\mathrm{t}\mathrm{m}\mathrm{a}\mathrm{x}\left(\frac{Q{K}^{T}}{\sqrt{{d}_{k}}}\right){V} , $

where $ \mathrm{X}\in {\mathbb{R}}^{l\times d} $ denotes the input; $ \mathrm{Q}\in {\mathbb{R}}^{l\times {d}_{k}}$, $\mathrm{K}\in {\mathbb{R}}^{l\times {d}_{k}} $, $\mathrm{V}\in {\mathbb{R}}^{l\times {d}_{v}} $ denote the query, key and value transformed from the input; $ {W}^{\,Q}\in {\mathbb{R}}^{d\times {d}_{k}},{W}^{\,K}\in {\mathbb{R}}^{d\times {d}_{k}},{W}^{\,V}\in {\mathbb{R}}^{d\times {d}_{v}} $ are the parameters to be learned.

Multi-head attention is based on “Scaled Dot-Product Attention” to increase the representation capability of the model by mapping the input sequences to different attention spaces:

$ \mathrm{M}\mathrm{u}\mathrm{l}\mathrm{t}\mathrm{i}\mathrm{H}\mathrm{e}\mathrm{a}\mathrm{d}\left(Q,K,V\right)=Concat\left({head}_{1}, \dots ,{head}_{h}\right){W}^{\,o} , $

$ {\mathrm{Where}}\; {head}_{i}=Attention(Q{W}_{i}^{\,Q},K{W}_{i}^{\,K},V{W}_{i}^{\,V}) , $

where $ {W}_{i}^{\,Q}\in {\mathbb{R}}^{d\times {d}_{k}},{W}_{i}^{\,K}\in {\mathbb{R}}^{d\times {d}_{k}},{W}_{i}^{\,V}\in {\mathbb{R}}^{d\times {d}_{v}},{W}^{\,o}\in {\mathbb{R}}^{h{d}_{v}\times d} $ are the parameters to be learned.

### BERT architecture

BERT (Devlin *et al*. 2019) is a multi-layer bidirectional language model obtained by stacking Transformer's (Vaswani *et al*. 2017) encoders. As shown in Fig. 1B, given a sequence containing L words $ \mathrm{X}=\{{x}_{1},{x}_{2},\dots ,  {x}_{L- 1},{x}_{L}\} $, recovering the content of the masked portion of the sequence is the training objective of BERT. Using the *i*^th^ word masked as an example, then BERT is trained with the training objective of maximizing the following likelihood: $ \mathrm{p}({M}_{i}={x}_{i}|{x}_{1}\dots {x}_{i- 1},{x}_{i+ 1},\dots {x}_{L}) $, where $ {M}_{i} $ denotes the word predicted by BERT after masking the *i*^th^ word of that.

### GPT architecture

GPT (Radford *et al*. 2018, 2019) is a multi-layer and unidirectional language model obtained by stacking the Transformer's decoder, and similar to the encoder introduced in the previous section, each decoder also consists of a multi-head attention module and a forward propagation module. Predicting the next word from the previous text is the training objective of GPT (see Fig. 1C), and the multi-head attention layer ensures that GPT can consider all the previous text when making predictions. Given a sequence containing L words $ \mathrm{X}=\{{x}_{1},{x}_{2},\dots ,{x}_{L- 1},{x}_{L}\} $, then GPT is trained with the training objective of maximizing the following likelihood: $ \mathrm{p}({N}_{i+ 1}={x}_{i+ 1}|{x}_{1}\dots {x}_{i- 1},{x}_{i}) $, where $ {N}_{i+ 1} $ denotes the (*i* + 1)^th^ word predicted by GPT after considering the previous *i* words.

## LANGUAGE MODELS FOR PROTEINS

**Figure 2:**
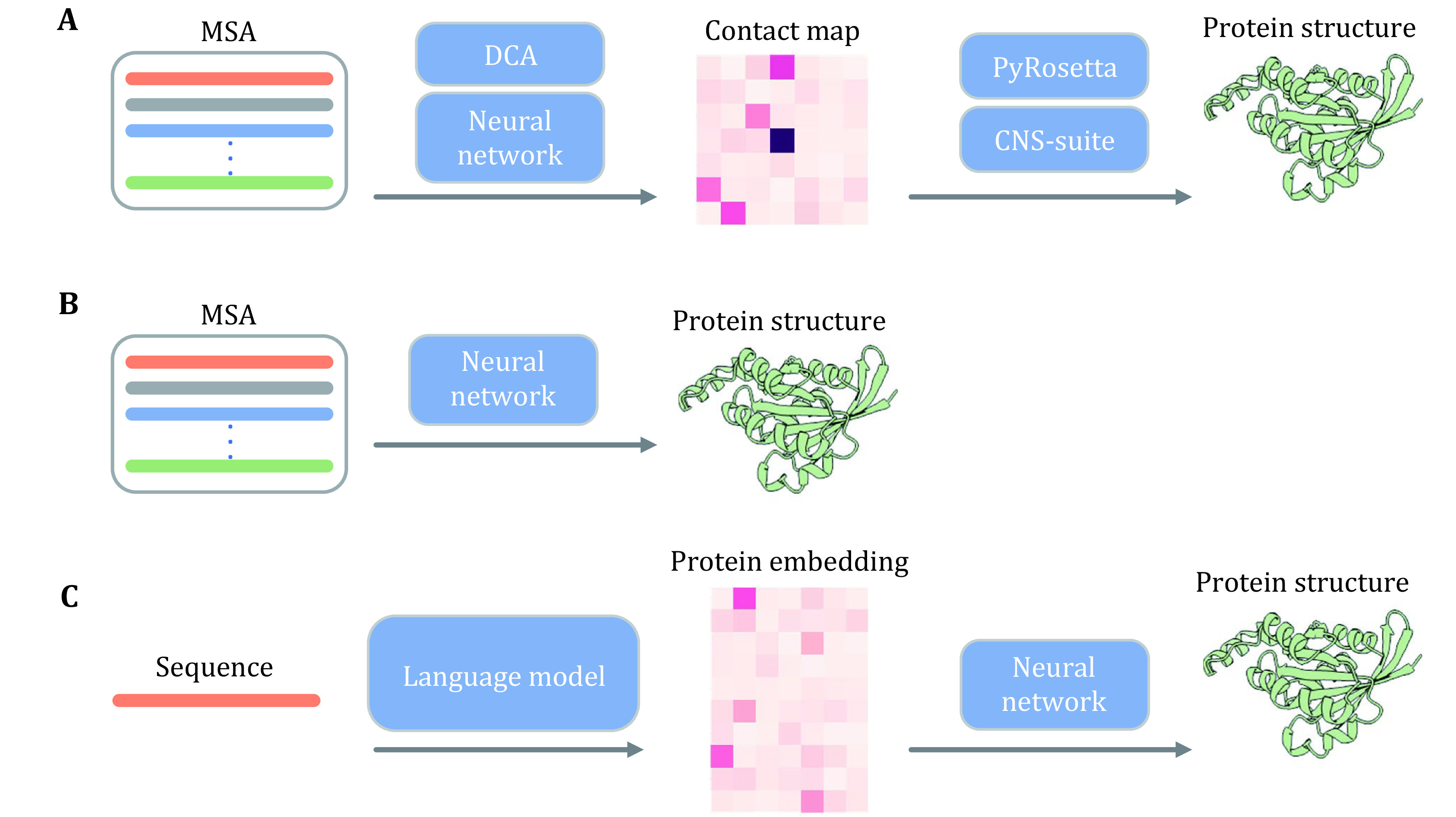
Frameworks for protein structure prediction. **A** Traditional paradigm for protein structure prediction. **B** MSA-based end-to-end protein structure prediction. **C** Protein structure prediction from a single sequence based on protein language model

Proteins are biological macromolecules composed of hundreds or thousands of amino acids (amino acids within proteins are often referred to as residues due to dehydration condensation), and the interactions between residues drive the folding of proteins into specific structures, which in turn perform specific functions (Kim *et al*. 2014). Given the importance of protein structure, countless approaches have been proposed over the past decades to advance the problem (Ding *et al*. 2018; Golkov *et al*. 2016; He *et al*. 2017; Jones *et al*. 2015; Ju *et al*. 2021; Wang *et al*. 2017; Xu 2019; Yang *et al*. 2020). Among them, the approaches that utilize mutual information, direct coupling analysis, and other tools to derive residue interactions from multi-sequence comparisons, and to predict protein structure from residue interactions using tools such as PyRosetta (Chaudhury *et al*. 2010), CNS (Brunger 2007), and others, have achieved remarkable success and have become the dominant paradigm for protein structure prediction (Senior *et al*. 2020; Wang *et al*. 2017; Yang *et al*. 2020). The methods of predicting residue interactions with the help of deep learning such as residue network (He *et al*. 2016) are the latest advances in this paradigm, but they are still far from solving the problem of protein structure prediction, while the introduction of language models has pushed the problem of protein structure prediction to be basically solved (Baek *et al*. 2021; Jumper *et al*. 2021; Lin *et al*. 2023) (the paradigms for protein structure prediction are illustrated in Fig. 2). Protein language models trained with a large number of protein sequences are able to capture the interactions between residues in protein sequences very well, and have already demonstrated very powerful capabilities in other downstream tasks such as protein structure prediction and protein function prediction. In addition to protein understanding, protein language models have also demonstrated excellent generative capabilities, which are very important for protein design problems such as protein sequence generation. We introduce protein language models below, which are focused on protein understanding (protein sequence modeling) and protein sequence generation.

### Protein sequence modeling based on protein language model

Sequence modeling has been a long-standing research problem in the domain of natural language processing, and advances in the NLP domain have shown that language models trained on huge amounts of unlabeled sequences, especially those based on the Transformer architecture, have a very good ability to model sequences. This success quickly extended to other research domains, and protein science was a pioneer in applying language models. Early protein language models for protein sequence modeling were mainly trained on protein sequence datasets in the form of predicting the content of the masked portion, SeqVec (Heinzinger *et al*. 2019) was trained using LSTM-based neural networks, and the analysis showed that the protein representations obtained from SeqVec were able to characterize the stability of proteins very well. TAPE (Rao *et al*. 2019) respectively trained language models using the mainstream CNN (Convolutional neural network) (Lecun *et* *al*. 1998), LSTM, and Transformer as the backbone networks, and proved that the Transformer-based language models had better performance compared to other architectures; ESM-1b (Rives *et al*. 2021) increases the number of parameters of the model by about 17 times from TAPE-Transformer by widening and deepening the number of network layers, and changes the training set from the Pfam (Mistry *et al*. 2021) protein sequence database used for training TAPE-Transformer to the Uniref50 (Mirdita *et al*. 2017) protein sequence database, and the analysis results show that ESM-1b The analysis results show that ESM-1b significantly outperforms TAPE-Transformer in the core task of capturing residue interactions (residue contact prediction), and also outperforms TAPE-Transformer in downstream tasks such as protein stability prediction and secondary structure prediction, which makes ESM-1b one of the most widely used protein language models. In ProtTrans's work (Elnaggar *et al*. 2022), the effect of language model architecture and sequence database size on the performance of protein language models was investigated by using multiple architectures of language models trained on a variety of different protein sequence databases, and the analysis results showed that the protein language model with the T5-XL (Raffel *et al*. 2019) architecture trained on the Uniref50 protein sequence dataset slightly outperforms ESM-1b on downstream tasks, such as secondary structure prediction, protein subcellular localization prediction, *etc*. Accurate protein structure prediction is a long-standing challenge in protein science, especially for single-sequence proteins. Given the excellent ability of protein language models to capture residue interactions, trRosettaX-Single (Wang *et al*. 2022), RGN2 (Chowdhury *et al*. 2022), EMBER2 (Ben-Tal and Kolodny 2022), OmegaFold (Wu *et al*. 2022 ), ESMFold (Lin *et al*. 2023), *etc*. attempted to realize accurate single sequence protein structure prediction with protein language models, these methods not only surpass the traditional "MSA-Contact/Distance-Structure" paradigm in terms of prediction speed, but also have a certain prediction ability for orphan proteins without homologous sequences. In addition to protein structure prediction, LMSuccSite (Pokharel *et al*. 2022) applied protein language models to Protein Succinylation Sites Prediction, IDP-LM (Pang and Liu 2023) applied protein language models to protein intrinsic disorder prediction, DeepGOPlus (Kulmanov and Hoehndorf 2020) applied protein language models to protein function prediction, and all achieved favorable results.

Compared with single protein sequences, homologous sequences in multiple sequence alignments contain rich evolutionary information that can greatly assist the inference of residue interactions; therefore, compared with protein language models based on single protein sequences, protein language models based on multiple sequence alignments may be more capable of capturing residue interactions. MSA-Transformer (Rao *et al*. 2021) is, to the best of our knowledge, the first protein language model trained based on multiple sequence alignment, which is built primarily from axis-attention based modules and is also trained with the objective of recovering the content of masked regions. The analysis results show that MSA-Transformer significantly outperforms ESM-1b in capturing residue interactions and achieves the best performance on the task of protein residue contact prediction. A-Port (Hong *et al*. 2022) performs residue contact prediction using MSA-Transformer and inputs the predicted pairs of contacting residues into PyRosetta for protein structure prediction. The analysis results show that the quality of structures predicted by A-Port exceeds the current best structure prediction methods, but it is still far from solving the problem of protein structure prediction. The emergence of AlphaFold2 (Jumper *et al*. 2021) has virtually solved the problem of structure prediction for proteins, and results at CASP14 show that for most proteins, the quality of the structure predicted by AlphaFold2 is comparable to the quality of the experimentally resolved structure. AlphaFold2 is a protein language model in an encoder-decoder architecture, where the encoder consists of a stack of 48 EvoFormer modules to extract the representation of multiple sequence alignments and explicitly predict the spatial distance between residues. The decoder, or structure module, consists of eight layers stacked on top of each other, which is used to generate the protein structure from the MSA representation. Specifically, the decoder initializes the spatial position of each residue in the protein at the origin, and each subsequent layer updates the protein structure with the sequence representations and residue distances from the encoder.

### Protein sequence generation based on protein language model

Generating protein sequences from scratch and generating constraint-compliant protein sequences are the two main application scenarios for protein sequence generation. Currently, although the Uniref100 protein sequence database (Mirdita *et al*. 2017) already contains about 250 million protein sequences, these protein sequences only account for a very small portion of the protein sequence space, so if foldable protein sequences can be generated computationally and rapidly, it can provide more options for fields that can use proteins, such as catalysis or pharmaceuticals, *etc*. ProtGPT2 (Ferruz *et al*. 2022) is a protein language model trained on 45 million protein sequences with the training goal of predicting the next word based on the current sentence. The training goal of ProtGPT2 makes ProtGPT2 naturally suitable for generating protein sequences from scratch. Analysis of the protein sequences predicted by ProtGPT2 showed that the proportion of disordered structures and amino acid frequencies are almost the same as the natural sequences, indicating that ProtGPT2 has the ability to generate protein sequences similar to the natural protein sequences. RITA (Hesslow *et al*. 2022) explored the effect of the scale of protein language model on the generative ability by training a series of protein language models of different scales with the objective of the next word prediction, and the results showed that the larger the scale of the language model, the higher the reliability of the generated protein sequence. In addition to this, Robert *et al*. (Verkuil *et al*. 2022) also explored the use of a masked protein language model to generate protein sequences and experimentally verified that the generated sequences have a higher probability (67%) of being soluble. ProtGen (Madani *et al*. 2023) is a representative work in generating protein sequences under finite constraints, which is also a protein language model with the training objective of predicting the next word. Compared with other protein language models, ProtGen can specify the function of the protein and then generate protein sequences that match the function, and experiments show that the protein sequences generated by ProtGen can realize some functions better than natural sequences and have lower similarity with existing natural protein sequences.

## LANGUAGE MODELS FOR GENOMICS

DNA and RNA are also important biomacromolecules in organisms like proteins. DNA mainly serves to encode genetic information, and interpreting DNA with the help of language modeling is a field of research that has emerged in the last two years; whereas for RNA only about 5% of all RNA transcripts are mRNAs coding for proteins, the remaining portion called non-coding RNAs exercise functions such as signaling and gene regulation, *etc*. (Wang and Chang 2011). Non-coding RNAs can perform specific functions only if they can maintain specific structures, but the severe scarcity of RNA structural data in the field of RNA has limited the performance of RNA structure prediction methods. In contrast to structural data, RNA sequence data has been accumulated with the development of RNA sequencing technology, and the structure of RNA is determined by the interactions between nucleotides, so how to distill the interactions between nucleotides with the help of the huge amount of RNA sequence data has become an important issue, and this is the area where language modeling specializes in.

**Figure 3:**
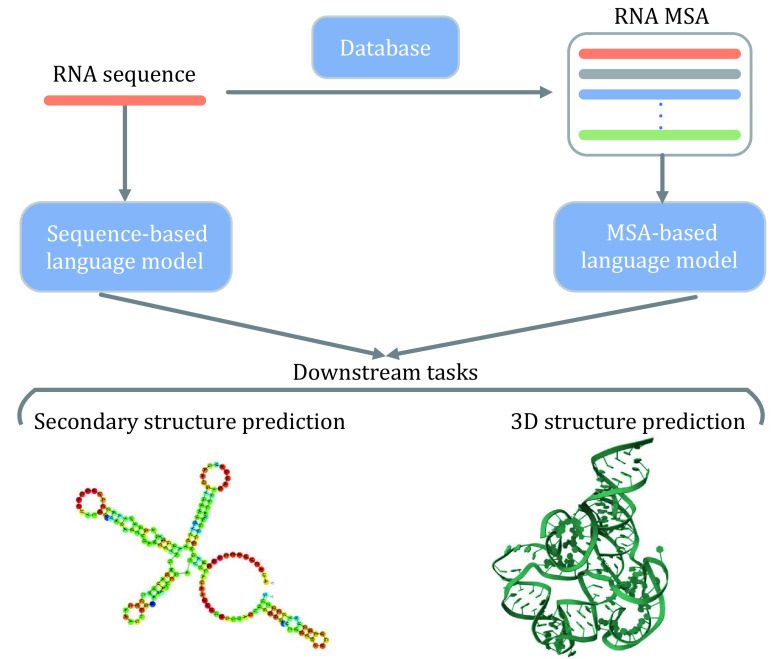
Applications of foundation models in RNA science. Both sequence-based and MSA-based RNA foundation models can be applied to downstream tasks such as RNA secondary structure prediction, RNA structure prediction, *etc.*

Developing DNA language models and RNA language models are rising research areas, the development of DNA/RNA language models as well as their applications will be described below (see Fig. 3).

### DNA sequence modelling based on the DNA language model

DNABERT (Ji *et al*. 2021) is, to the best of our knowledge, the first DNA language model using the BERT architecture, specifically, DNABERT uses the human genome as the training data and the *k*-mer representation of DNA as words for training (Take the DNA sequence “ATGGCT” as an example, the 3-mer representation used by DNABERT will represent the sequence as {ATG, TGG, GGC, GCT}). The excellent performance of DNABERT in predicting proximal and core promoter regions and identifying transcription factor binding sites fully demonstrates the potential of language models in the field of DNA research. In contrast to DNABERT, which was trained using only the human genome, Nucleotide Transformer (Dalla-Torre *et al*. 2023) was trained using the genomes of 850 species and showed excellent performance in detecting genetic variants and predicting the effects of mutations. DNABERT-2 (Zhou *et al*. 2023b) is an upgraded version of DNABERT, which not only proposes a simple and effective scheme for DNA tokenization, but also dramatically improves the training efficiency by adopting techniques such as Flash Attention. In addition, representative work using a DNA foundation model for CRISPR sgRNA design, *i*.*e*., DeepCRISPR (Chuai *et al*. 2018), was presented.

### Non-coding RNA sequence modelling based on RNA language model

RNA-FM (Chen *et al*. 2022) adopts the BERT architecture and uses twenty-three million non-coding RNAs from RNAcentral (The RNAcentral Consortium 2019) for training, which is trained by randomly masking a portion of the RNA sequence and then aiming to recover the content of the masked region. The analysis results on downstream tasks such as nucleotide distance prediction, secondary structure prediction, *etc*. show that the prediction performance using RNA-FM is better than that using only RNA sequences, suggesting that RNA-FM captures partial nucleotide interactions. Uni-RNA (Wang *et al*. 2023e) also employs the BERT architecture and trains with the goal of recovering the contents of the masked region, but the training set of Uni-RNA contains 1 billion non-coding protein sequences from databases such as nt (NCBI Resource Coordinators 2014), RNAcentral (The RNAcentral Consortium 2019), Genome Warehouse (GWH) (Chen *et al*. 2021a), and others. Test results on tasks such as nucleotide contact prediction show that Uni-RNA outperforms RNA-FM across the board, indicating that Uni-RNA has a stronger ability to capture nucleotide interactions.

Compared to single sequences, there are also some RNA language models developed based on the MSA of RNA. RNA-MSM (Zhang *et al*. 2023) adopts the MSA-Transformer architecture and uses 3932 MSAs for training, and outperforms traditional algorithms in water solubility prediction as well as secondary structure prediction tasks, which proves the application value of RNA language model. In addition, works such as trRosettaRNA (Wang *et al*. 2023d), DRfold (Li *et al*. 2023), and RoseTTAFoldNA (Baek *et al*. 2024) used a similar architecture to the encoder of AlphaFold2 to process MSA for RNA structure prediction, and also achieved certain results.

## LANGUAGE MODELS FOR SINGLE CELL TRANSCRIPTOMES

**Figure 4:**
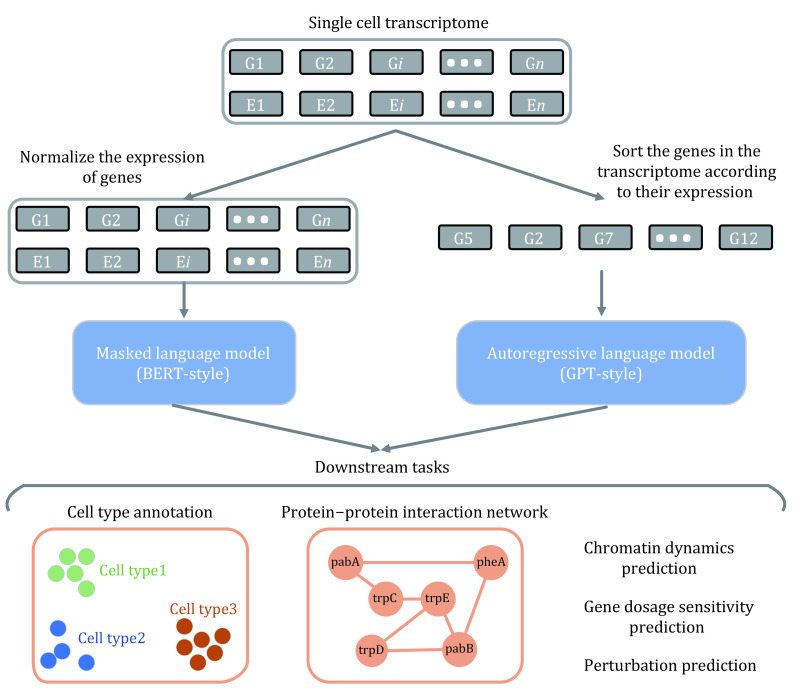
Applications of foundation models in single cell transcriptomes. BERT-style and GPT-style foundational models take different forms of single cell transcriptome data as input and can be applied to downstream tasks such as cell type annotation and chromatin dynamics prediction, where G denotes gene and E denotes expression

Cells are the basic units of life, the complex regulatory relationships between intracellular genes determine the behavior and function of cells, and the complex interactions between various types of cells in an organism realize more advanced life activities. Deciphering the intracellular regulatory network between genes and the communication network between cells in an organism is extremely crucial for analyzing the differences between different types of cells and understanding the life process, and the development of single-cell transcriptome sequencing technology has dramatically advanced this process (Kolodziejczyk *et al*. 2015; Jovic *et al*. 2022). The transcriptome is the total of the transcription products of all genes in a cell under specific spatial and temporal conditions, which determines the specificity of the cell, and it is also the result of complex intra- and inter-cellular regulatory relationships. Single cell transcriptome sequencing technology has accumulated a large amount of single cell transcriptome data in the past decade (Cao *et al*. 2017; Moreno *et al*. 2022), and there are numerous algorithms tried to decipher the mystery of intracellular gene regulation and intercellular communication with the help of single cell transcriptome data (Bafna *et al*. 2023; Dai *et al*. 2019; Iacono *et al*. 2019; Wang *et al*. 2023c). Recently, transcriptome language models have made great progress in capturing gene regulatory relationships (Cui *et al*. 2023; Theodoris *et al*. 2023; Wen *et al*. 2023; Yang *et al*. 2022), and have gradually become the main method to analyze single cell transcriptome data (see Fig. 4). In addition, transcriptome language models have also shown very good performance in cell type identification, gene expression prediction and other tasks. In the following, we will introduce the training approaches and applications of transcriptome language models.

The transcriptome of a single cell contains both gene types and corresponding gene expressions, an ideal transcriptome language model should have the ability to capture the causal relationships between all elements (gene types, gene expressions) in the transcriptome, while the ability of the model is closely related to the design of the model's training objective. Earlier transcriptome language models were mainly trained based on recovering the content of the masked region as the training objective, but there are some differences in the way of masking. scBERT (Yang *et al*. 2022) uses the Performer module to build the model, which is capable of handling longer sequences than the standard Transformer. In addition, scBERT was trained using the Panglao human single-cell transcriptome dataset (containing about one million transcriptomes) by masking a portion of the expression of a gene in the transcriptome (with non-zero expression) and then predicting the expression of the masked portion. scBERT achieves the best performance on the tasks of cell type annotation and identification of novel cells, which indicates that the model captures cell specificity. Compared to scBERT, which only aims at recovering the gene expression in the masked region, scFormer's (Cui *et al*. 2022) training objective includes both recovering the gene expression in the masked region and recovering the gene type in the masked region, and it also achieves good performance on tasks such as gene perturbation as well as batch effect correction. Gene expression can fluctuate widely, and gene expression can also contain overall noise due to batch effects, *etc*. Geneformer (Theodoris *et al*. 2023) has designed a new type of training objective to train the transcriptome language model, specifically, Geneformer will first sort the genes in the transcriptome according to their expression, and then, after masking the genes randomly, it will set the training objective to predict the types of genes at the masked positions, which cleverly uses the information of genes and expressions, and also eliminates the noise problem in the expressions. The analysis results show that Geneformer can handle batch effects well and performs well on tasks such as network dynamics prediction as well as gene perturbation prediction, suggesting that Geneformer learns the regulatory relationships between genes from the transcriptome well. scFoundation (Hao *et al*. 2023) considers that the vast majority of genes in the single-cell transcriptome are not expressed (expression is zero), and complete processing of all genes and expression will greatly affect the inference speed of the model as well as the scale of the trainable model; therefore, an asymmetric encoder-decoder language model architecture was designed, in which the encoder module only processes genes with an expression not zero. This architecture allows scFoundation to reach a scale of 100 million parameters and outperforms pre-trained models such as scBERT and Geneformer.

In addition to transcriptome language models that are trained with the objective of recovering the content of masked regions, work such as scGPT (Cui *et al*. 2023) as well as scTranslator (Liu *et al*. 2023) have explored the application of generative language models in the transcriptome. scGPT is trained to sequentially predict the expression of genes with unknown expression based on the known gene expression and cell type, and thus the model has the ability to generate the transcriptome of an entire cell while only the cell type is specified. scTranslator, on the other hand, is a generative transcriptome language model trained to infer protein abundance values. scTranslator can predict the proteome of a single cell given that cell's transcriptome, and analysis has shown that the interactions between proteins (genes) inferred by scTranslator are relatively accurate.

## GRAPH NEURAL NETWORKS ON SPATIAL TRANSCRIPTOMICS

Recent advances in spatially-resolved transcriptomics (ST) technologies have enabled telescoped investigation of *in situ* gene expression and spatial location of cells in tissues. The spatial transcriptomics data profiles cell type structure, gene expression with spatial pattern and cell-to-cell interactions in spatial perceptions. This knowledge is essential for understanding and explaining complex life systems, *i*.*e*., disease progress (Ye *et al*. 2022; Chen *et al*. 2020), tumor micro-environment (Zhu *et al*. 2022; Ferri-Borgogno *et al*. 2023) and organogenesis (Chen *et al*. 2022). Generally, ST technologies can be commonly classified into two categories. The first category is image-based technologies such as *in situ* hybridization *in situ* sequencing, which includes seqFISH (Shah *et al*. 2018), MERFISH (Zhang *et al*. 2021) and STARmap (Wang *et al*. 2018). The second category is capture and sequencing-based technology, which includes 10x Visium (Wang *et al*. 2021), Slide-Seq (Rodriques *et al*. 2019), Slide-Seq2 (Stickels *et al*. 2021), HDST (Vickovic *et al*. 2019) and Stereo-seq (Chen *et al*. 2022). These ST technologies have been well utilized in multiple organisms, *i*.*e*., human, mouse, and drosophila.

**Figure 5:**
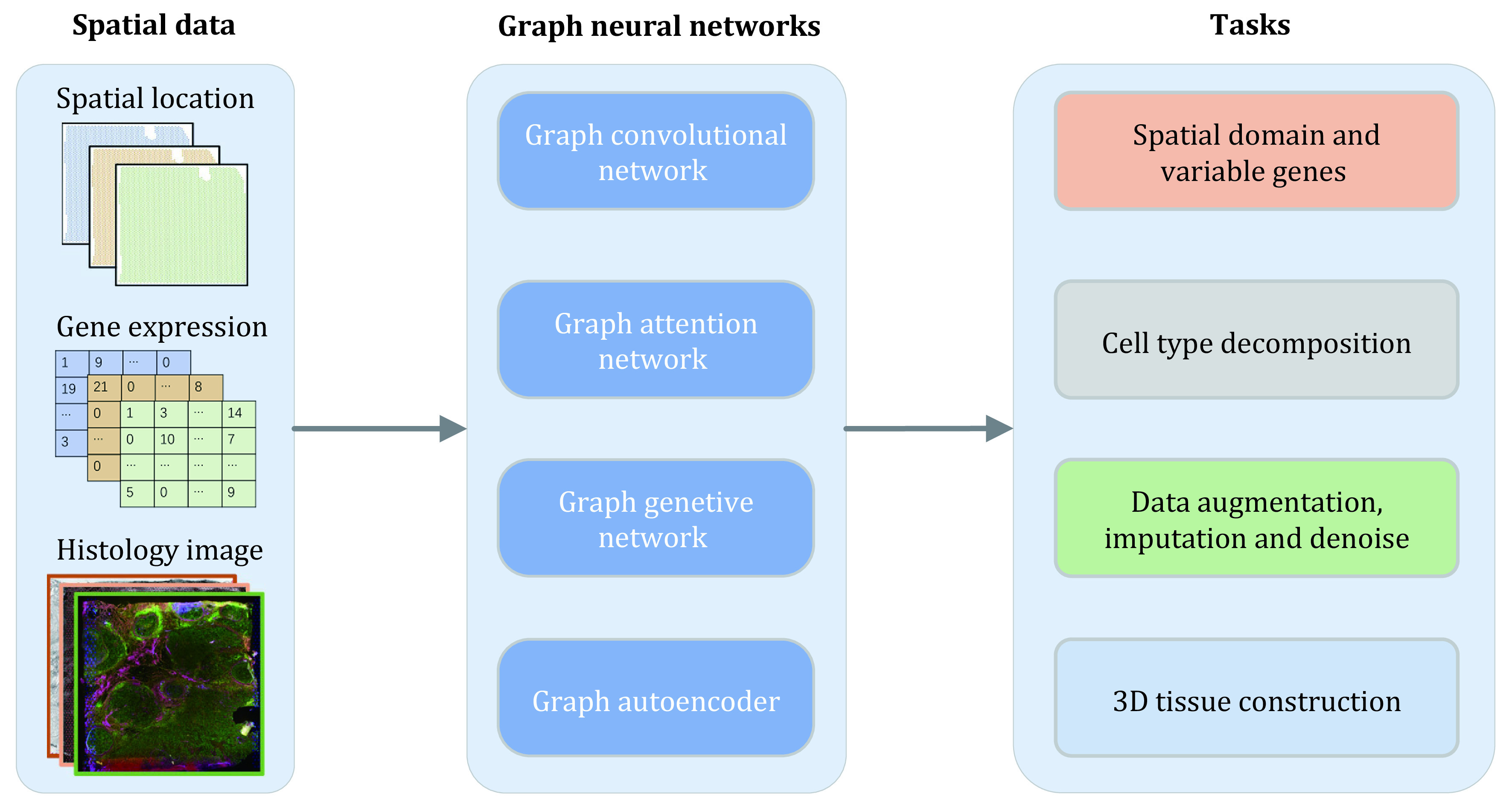
Overview of graph neural networks on spatial transcriptomics

Although ST provides revolutionized data of tissue, it’s challenged by barriers from intrinsic noise, high-sparseness, and multimodality (gene expression matrices, spatial coordinates and histology images). The main task of analyzing ST datasets includes the detection of spatial domain and variable genes (SVGs), cell type decomposition and data augmentation. Besides, three-dimensional (3D) cellular structure construction is required to better understand the biological process in the whole organ and organism. In order to accomplish these needs, lots of computational methods have been developed. Graph neural networks (GNNs) have attracted much attention in recent articles (Wu *et al*. 2019; Liu *et al*. 2024). Unlike other common methods which failed to utilize the spatial coordinates and histology image information, GNNs enable learning from a bucket of gene expression data, spot spatial coordinates, *i*.*e*., graph neighborhood network, and histology image. GNNs are generally self-supervised or semi-supervised models, as shown in Fig. 5, the GNNs utilized in ST methods can be generally divided into four categories, *i*.*e*., graph convolutional network (GCN), graph attention network (GAN), graph generative network and graph autoencoder. Compared with other models, these GNNs can learn and preserve the relative information in spatial location and image data, which makes them outperform in many tasks such as spatial domain detection, cell type decomposition and 3D tissue construction.

As mentioned before, due to the low capture efficiency and high technology noise in ST data, data augmentation (imputation, denoise) is a key task in ST data analysis. For this task, one kind of method is to integrate scRNA-seq data with ST, such as stPlus (Chen *et al*. 2021b) and SpaGE (Abdelaal *et al*. 2020). However, doing so might induce new bias and unwanted noise due to the unpaired samples and technology differences. Another kind of method mainly considers the ST data itself and usually makes the augmentation with the neighborhood structure of ST spots, which is associated with spatial location. In this situation, GNN-based methods can be appealing, *i*.*e*., SEDR (Fu *et al*. 2021), stMVC (Zuo *et al*. 2022) and SiGra (Tang *et al*. 2023). SEDR is an unsupervised model that integrates transcriptomics data and associated spatial information. It first constructs a low-dimension latent representation of the ST matrix through a deep autoencoder, and then combines it with the corresponding spatial loci information by a variational graph autoencoder. The SEDR pipeline performed well on human dorsolateral prefrontal cortex data, and was able for batch correction. stMVC is a muti-modal model method that integrates gene expression matrix, spatial location, histology image and region segmentation. It applied a semi-supervised graph attention autoencoder to capture the structure of ST data, and the whole model can elucidate intra-tumoral heterogeneity in ST data. SiGra was designed to denoise gene expression data in ST. A graph transformer was used to leverage the rich information in the spatial distribution of spots and cells, and the inclusion of immunohistochemistry images by imaging-transcriptomics hybrid architecture can help improve the performance by 37%.

Deciphering spatial domains and SVGs is critical for understanding the biological structure and function of tissue. In this task, models must consider the spatial location of cells and gene expression. SpaGCN (Hu *et al*. 2021) applied a graph convolutional network (GCN)-based approach to detect spatial domain and SVGs. The spatial domain detection is based on the weighted graph built on gene expression and histology image and spatial location, and then SVGs are calculated on spatial domains. STAGATE (Dong and Zhang 2022) developed a graph attention autoencoder framework to identify spatial domains. The graph attention autoencoder learns to integrate gene expression and spatial location information, and adopts a graph attention mechanism when considering spatial neighbor information. STAGATE performed well in the accuracy of spatial domain and SVGs detection. CCST (Li *et al*. 2022a) is an unsupervised cell clustering method based on GCN. The cell cluster results provided by CCST can help identify curate cell type and then spatial domain. Spatial-MGCN (Wang *et al*. 2023a) adopted a multi-view GCN encoder to extract unique embeddings from gene expression and spatial location graphs. The incorporation of this information in Spatial-MGCN helps it outperform in spatial domain detection.

The resolution of the majority ST technologies has not reached a single-cell level, thus decomposition of cell type in ST data is commonly needed. There are lots of methods designed for ST cell type decomposition utilizing scRNA-seq as a reference, *i*.*e*., cell2location (Kleshchevnikov *et al*. 2022), SPOTlight (Elosua-Bayes *et al*. 2021) and Tangram (Biancalani *et al*. 2021). The spatially nearby spots are more likely to share similar cell components, thus leveraging spatial location by GNNs could improve cell-type decomposition performance. DSTG (Song and Su 2021) adopts GCN to learn the latent representation of both gene expression and spatial locations of spots, and later applied decomposition on the latent representation matrix. GraphST (Long *et al*. 2023) is a graph self-supervised contrastive learning method. A GNN accompanied by augmentation-based self-supervised contrastive learning is used to learn representations of spots in GraphST.

3D construction of whole tissue or organs can accelerate the understanding of disease processes and organogenesis. Since one individual ST slice contains gene expression information on a 2D plane, the 3D construction of tissue requires the integration of multiple slices. There are several methods for integrating parallel ST slices and 3D construction, *i*.*e*., PASTE (Zeira *et al*. 2022), STAligner (Zhou *et al*. 2023a) and Stihchi3D (Wang *et al*. 2023b). PASTE mainly aligns spots in different slices based on their gene expression similarity and spatial distances, using an optimal transport algorithm. STAligner develops a graph attention autoencoder to learn spot embeddings with gene expression and spatial location information. The later alignment is based on the embedding and shared spatial domain between slices. Stihchi3D is a joint model for 3D domain detection and cell-type decomposition of ST. A graph attention network is utilized to learn the representation of spots’ gene expression and 3D spatial adjacent network.

In summary, ST contains multi-modal data, *i*.*e*., gene expression, spatial locations and histology image, which requires full usage of this information. GNNs are efficient at capturing relative information from network-style data. While dealing with noisy and sparse ST data, GNNs have great potential in solving tasks including data augmentation, spatial domain and SVGs detection, cell type decomposition and 3D construction of tissue.

## DISCUSSION

Foundational models in molecular biology are shaping new research approaches in the field, in this review we provide a comprehensive summary of foundational models in molecular biology, detailing their architecture, training approaches, scope of application, and how they are used. Noted that although significant achievements have been made by foundational models in molecular biology, most current language models are based on specific types of biological data, and cross-modal foundational models of greater value are still relatively rare. Another important issue regarding the foundation model is its relationship with “small sample learning”, *i*.*e*. the few-shot learning using relatively small training samples (Long *et al*. 2023). It should be noted that the “fine-tuning” strategy used in the foundation model is actually targeted to address the small sample issue in the specific downstream tasks. However, a recent study indicated that the foundation model may fail in the zero-shot scenario, which is an extreme case of few-shot learning (Zeira *et al*. 2022) in which no training data are available for the specific tasks. For such low-data-resource learning cases, various few-shot learning schemas, for example, the meta learning has been proposed (Zhou *et al*. 2023a). Several applications using meta learning to address molecule analysis problems, for example, the pMHC-TCR interaction recognition (Wang *et al*. 2023b) and kinome-wide polypharmacology profiling have been presented (Benegas *et al*. 2023).

Finally, life processes are often dynamic, and multi-modal foundational models that can take into account the spatio-temporal specificity of biological data may be able to make the digital cell a reality.

## Conflict of interest

Yunda Si, Jiawei Zou, Yicheng Gao, Guohui Chuai, Qi Liu and Luonan Chen declare that they have no conflict of interest.

## References

[bAbdelaal2020] (2020). SpaGE: spatial gene enhancement using scRNA-Seq. Nucleic Acids Res.

[bBaek2021] (2021). Accurate prediction of protein structures and interactions using a three-track neural network. Science.

[bBaek2024] (2024). Accurate prediction of protein–nucleic acid complexes using RoseTTAFoldNA. Nat Methods.

[bBafna2023] (2023). CLARIFY: cell–cell interaction and gene regulatory network refinement from spatially resolved transcriptomics. Bioinformatics.

[bBai2015] (2015). How Cryo-EM is revolutionizing structural biology. Trends Biochem Sci.

[bBenegas2023] (2023). DNA language models are powerful predictors of genome-wide variant effects. Proc Natl Acad Sci USA.

[bBenTal2022] (2022). Homologues not needed: structure prediction from a protein language model. Structure.

[bBepler2021] (2021). Learning the protein language: evolution, structure, and function. Cell Systems.

[bBiancalani2021] (2021). Deep learning and alignment of spatially resolved single-cell transcriptomes with tangram. Nat Methods.

[bBrown2020] Brown TBMann B, Ryder N, Subbiah M, Kaplan JD, Dhariwal P, Neelakantan A, Shyam P, Sastry G, Askell A, Agarwal S, Herbert-Voss A, Krueger G, Henighan T, Child R, Ramesh A, Ziegler DM, Wu J, Winter C, Hesse C, Chen M, Sigler E, Litwin M, Gray S, Chess B, Clark J, Berner C, McCandlish S, Radford A, Sutskever I, Amodei D (2020) Language models are few-shot learners. In: Advances in Neural Information Processing Systems. pp. 1877–1901

[bBrunger2007] (2007). Version 1.2 of the crystallography and NMR system. Nat Protocols.

[bCao2017] (2017). scRNASeqDB: a database for RNA-Seq based gene expression profiles in human single cells. Genes (Basel).

[bChaudhury2010] (2010). PyRosetta: a script-based interface for implementing molecular modeling algorithms using Rosetta. Bioinformatics.

[bChen2022a] (2022). Spatiotemporal transcriptomic atlas of mouse organogenesis using DNA nanoball-patterned arrays. Cell.

[bChen2022b] Chen J, Hu Z, Sun S, Tan Q, Wang Y, Yu Q, Zong L, Hong L, Xiao J, Shen T, King I, Li Y (2022) Interpretable RNA foundation model from unannotated data for highly accurate RNA structure and function predictions. arXiv. https://doi.org/10.48550/arXiv.2204.00300

[bChen2021a] (2021). Genome warehouse: a public repository housing genome-scale data. Genomics, Proteomics Bioinformatics.

[bChen2021b] (2021). stPlus: a reference-based method for the accurate enhancement of spatial transcriptomics. Bioinformatics.

[bChen2020] (2020). Spatial transcriptomics and *in situ* sequencing to study Alzheimer’s disease. Cell.

[bChowdhury2022] (2022). Single-sequence protein structure prediction using a language model and deep learning. Nat Biotechnol.

[bChuai2018] (2018). DeepCRISPR: optimized CRISPR guide RNA design by deep learning. Genome Biol.

[bCirillo2012] (2012). Predictions of protein–RNA interactions. WIREs Comput Mol Sci.

[bCui2022] Cui H, Wang C, Maan H, Duan N, Wang B (2022) scFormer: a universal representation learning approach for single-cell data using transformers. bioRxiv. https://doi.org/10.1101/2022.11.20.517285

[bCui2023] Cui H, Wang C, Maan H, Pang K, Luo F, Duan N, Wang B (2023) scGPT: towards building a foundation model for single-cell multi-omics using generative AI. Nat Methods. https:// doi.org/10.1038/s41592-024-02201-0

[bCui2020] Cui Y, Che W, Liu T, Qin B, Wang S, Hu G (2020) Revisiting pre-trained models for Chinese natural language processing. In: Findings of the Association for Computational Linguistics: EMNLP 2020. pp. 657–668

[bDai2019] (2019). Cell-specific network constructed by single-cell RNA sequencing data. Nucleic Acids Res.

[bDallaTorre2023] Dalla-Torre H, Gonzalez L, Revilla JM, Carranza NL, Grzywaczewski AH, Oteri F, Dallago C, Trop E, Sirelkhatim H, Richard G, Skwark M, Beguir K, Lopez M, Pierrot T (2023) The nucleotide transformer: building and evaluating robust foundation models for human genomics. bioRxiv. https://doi.org/10.1101/2023.01.11.523679

[bDevlin2019] Devlin, Jacob, Ming-Wei Chang, Kenton Lee, and Kristina Toutanova (2019) BERT: Pre-Training of Deep Bidirectional Transformers for Language Understanding. In: Proceedings of the 2019 Conference of the North American Chapter of the Association for Computational Linguistics: Human Language Technologies, Volume 1 (Long and Short Papers). pp. 4171–4186

[bDing2018] (2018). DeepConPred2: An improved method for the prediction of protein residue contacts. Comput Struct Biotechnol J.

[bDobson1999] (1999). Protein misfolding, evolution and disease. Trends Biochem Sci.

[bDodge2020] Dodge J, Ilharco G, Schwartz R, Farhadi A, Hajishirzi H, Smith N (2020) Fine-tuning pretrained language models: weight initializations, data orders, and early stopping. arXiv. https://doi.org/10.48550/arXiv.2002.06305

[bDong2022] (2022). Deciphering Spatial domains from spatially resolved transcriptomics with an adaptive graph attention auto-encoder. Nat Commun.

[bDong2019] Dong L, Yang N, Wang W, Wei F, Liu X, Wang Y, Gao J, Zhou M, Hon H-W (2019) Unified language model pre-training for natural language understanding and generation. arXiv. https://doi.org/10.48550/arXiv.1905.03197

[bElnaggar2022] (2022). ProtTrans: towards cracking the language of lifes code through self-supervised deep learning and high performance computing. IEEE Trans Pattern Anal Mach Intell.

[bElosuaBayes2021] (2021). SPOTlight: seeded NMF regression to deconvolute spatial transcriptomics spots with single-cell transcriptomes. Nucleic Acids Res.

[bEthayarajh2019] Ethayarajh K (2019) How contextual are contextualized word representations? comparing the geometry of BERT, ELMo, and GPT-2 embeddings. In: Proceedings of the 2019 Conference on Empirical Methods in Natural Language Processing and the 9th International Joint Conference on Natural Language Processing (EMNLP-IJCNLP). pp. 55–65

[bFerriBorgogno2023] (2023). Spatial transcriptomics depict ligand-receptor cross-talk heterogeneity at the tumor-stroma interface in long-term ovarian cancer survivors. Cancer Res.

[bFerruz2022] (2022). ProtGPT2 is a deep unsupervised language model for protein design. Nat Commun.

[bFu2021] Fu H, Xu H, Chong K, Li M, Ang KS, Lee HK, Ling J, Chen A, Shao L, Liu L, Chen J (2021) Unsupervised spatially embedded deep representation of spatial transcriptomics. bioRxiv. https://doi.org/10.1101/2021.06.15.448542

[bGao2023] (2023). Hierarchical graph learning for protein–protein interaction. Nat Commun.

[bGolkov2016] Golkov, Vladimir, Marcin J. Skwark, Antonij Golkov, Alexey Dosovitskiy, Thomas Brox, Jens Meiler, and Daniel Cremers (2016) Protein contact prediction from amino acid co-evolution using convolutional networks for graph-valued images. In: Proceedings of the 30th International Conference on Neural Information Processing Systems. pp. 4222–4230

[bGoodsell2020] (2020). RCSB Protein Data Bank: enabling biomedical research and drug discovery. Protein Sci.

[bHao2023] Hao M, Gong J, Zeng X, Liu C, Guo Y, Cheng X, Wang T, Ma J, Song L, Zhang X (2023) Large scale foundation model on single-cell transcriptomics. bioRxiv. https://doi.org/10.1101/2023.05.29.542705

[bHartl2017] (2017). Protein misfolding diseases. Annu Rev Biochem.

[bHe2017] (2017). NeBcon: protein contact map prediction using neural network training coupled with naïve Bayes classifiers. Bioinformatics.

[bHe2020] (2020). Mask R-CNN. IEEE Trans Pattern Anal Mach Intell.

[bHe2016] He K, Zhang X, Ren S, Sun J (2016) Deep residual learning for image recognition. In: 2016 IEEE Conference on Computer Vision and Pattern Recognition (CVPR). pp. 770–778

[bHeinzinger2019] (2019). Modeling aspects of the language of life through transfer-learning protein sequences. BMC Bioinformatics.

[bHenderson2010] (2010). Molecular chaperones and protein-folding catalysts as intercellular signaling regulators in immunity and inflammation. J Leukoc Biol.

[bHesslow2022] Hesslow D, Zanichelli N, Notin P, Poli I, Marks D (2022) RITA: a study on scaling up generative protein sequence models. arXiv. https://doi.org/10.48550/arXiv.2205.05789

[bHong2022] (2022). A-Prot: protein structure modeling using MSA transformer. BMC Bioinformatics.

[bHu2021] (2021). SpaGCN: integrating gene expression, spatial location and histology to identify spatial domains and spatially variable genes by graph convolutional network. Nat Methods.

[bIacono2019] (2019). Single-cell transcriptomics unveils gene regulatory network plasticity. Genome Biol.

[bJankowsky2015] (2015). Specificity and nonspecificity in RNA–protein interactions. Nat Rev Mol Cell Biol.

[bJi2021] (2021). DNABERT: pre-trained bidirectional encoder representations from transformers model for DNA-language in genome. Bioinformatics.

[bJones2015] (2015). MetaPSICOV: combining coevolution methods for accurate prediction of contacts and long range hydrogen bonding in proteins. Bioinformatics (Oxford, England).

[bJoshi2018] Joshi V, Peters M, Hopkins M (2018) Extending a parser to distant domains using a few dozen partially annotated examples. arXiv. https://doi.org/10.48550/arXiv.1805.06556

[bJovic2022] (2022). Single-cell RNA Sequencing technologies and applications: a brief overview. Clin Transl Med.

[bJu2021] (2021). CopulaNet: learning residue co-evolution directly from multiple sequence alignment for protein structure prediction. Nat Commun.

[bJumper2021] (2021). Highly accurate protein structure prediction with AlphaFold. Nature.

[bKim2014] (2014). One contact for every twelve residues allows robust and accurate topology-level protein structure modeling. Proteins.

[bKlein2019] Klein T, Nabi M (2019) Learning to answer by learning to ask: getting the best of GPT-2 and BERT worlds. arXiv. https://doi.org/10.48550/arXiv.1911.02365

[bKleshchevnikov2022] (2022). Cell2location maps fine-grained cell types in spatial transcriptomics. Nat Biotechnol.

[bKolodziejczyk2015] (2015). The technology and biology of single-cell RNA sequencing. Mol Cell.

[bKulmanov2020] (2020). DeepGOPlus: improved protein function prediction from sequence. Bioinformatics.

[bLecun1998] (1998). Gradient-based learning applied to document recognition. Proc IEEE.

[bLenz2021] (2021). Reliable identification of protein-protein interactions by crosslinking mass spectrometry. Nat Communs.

[bLi2022a] (2022a). Cell clustering for spatial transcriptomics data with graph neural networks. Nat Comput Sci.

[bLi2014] (2014). starBase v2.0: decoding miRNA-ceRNA, miRNA-ncRNA and protein–RNA interaction networks from large-scale CLIP-seq data. Nucleic Acids Res.

[bLi2022b] (2022b). MARPPI: boosting prediction of protein–protein interactions with multi-scale architecture residual network. Briefings Bioinform.

[bLi2023] (2023). Integrating end-to-end learning with deep geometrical potentials for ab initio RNA structure prediction. Nat Commun.

[bLimo2018] (2018). Interactions between metal oxides and biomolecules: from fundamental understanding to applications. Chem Rev.

[bLin2023] (2023). Evolutionary-scale prediction of atomic-level protein structure with a language model. Science.

[bLiu2023] Liu L, Li W, Wong K-C, Yang F, Yao J (2023) A pre-trained large generative model for translating single-cell transcriptome to proteome. bioRxiv. https://doi.org/10.1101/2023.07.04.547619

[bLiu2024] (2024). A comprehensive overview of graph neural network-based approaches to clustering for spatial transcriptomics. Comput Struct Biotechnol J.

[bLong2023] (2023). Spatially informed clustering, integration, and deconvolution of spatial transcriptomics with GraphST. Nat Commun.

[bLu2020] (2020). Recent advances in the development of protein–protein interactions modulators: mechanisms and clinical trials. Signal Transduct Target Ther.

[bMadani2023] (2023). Large language models generate functional protein sequences across diverse families. Nat Biotechnol.

[bMann2017] (2017). IntaRNA 2.0: enhanced and customizable prediction of RNA–RNA interactions. Nucleic Acids Res.

[bMcDowall2009] (2009). PIPs: human protein–protein interaction prediction database. Nucleic Acids Res.

[bMirdita2017] (2017). Uniclust databases of clustered and deeply annotated protein sequences and alignments. Nucleic Acids Res.

[bMistry2021] (2021). Pfam: the protein families database in 2021. Nucleic Acids Res.

[bMoreno2022] (2022). Expression atlas update: gene and protein expression in multiple species. Nucleic Acids Res.

[bNCBI2014] (2014). Database resources of the national center for biotechnology information. Nucleic Acids Rese.

[bNguyen2016] (2016). Mapping RNA–RNA interactome and RNA structure *in vivo* by MARIO. Nat Commun.

[bNooren2003] (2003). Diversity of protein–protein interactions. EMBO J.

[bOughtred2021] (2021). The BioGRID database: a comprehensive biomedical resource of curated protein, genetic, and chemical interactions. Protein Sci.

[bPang2023] (2023). IDP-LM: prediction of protein intrinsic disorder and disorder functions based on language models. PLoS Computat Biol.

[bPeters2018] Peters ME, Neumann M, Iyyer M, Gardner M, Clark C, Lee K, Zettlemoyer L (2018) Deep contextualized word representations. In: Proceedings of the 2018 Conference of the North American Chapter of the Association for Computational Linguistics: Human Language Technologies, Vol. 1 (Long Papers). pp. 2227–2237

[bPokharel2022] (2022). Improving protein succinylation sites prediction using embeddings from protein language model. Sci Rep.

[bPuton2012] (2012). Computational methods for prediction of protein–RNA interactions. J Struct Biol.

[bRadford2018] Radford A, Narasimhan K, Salimans T, Sutskever I (2018) Improving language understanding by generative pre-training. https://openai-assets.s3.amazonaws.com/research-covers/language-unsupervised/language_understanding_paper.pdf

[bRadford2019] Radford A, Wu J, Child R, Luan D, Amodei D, Sutskever I (2019) Language models are unsupervised multitask learners. https://cdn.openai.com/better-language-models/language_models_are_unsupervised_multitask_learners.pdf

[bRaffel2019] Raffel C, Shazeer N, Roberts A, Lee K, Narang S, Matena M, Zhou Y, Li W, Liu PJ (2019) Exploring the limits of transfer learning with a unified text-to-text transformer. arXiv. https://doi.org/10.48550/arXiv.1910.10683

[bRamanathan2019] (2019). Methods to study RNA–protein interactions. Nat Methods.

[bRao2019] (2019). Evaluating protein transfer learning with TAPE. Adv Neural Inf Process Syst.

[bRao2021] Rao RM, Liu J, Verkuil R, Meier J, Canny J, Abbeel P, Sercu T, Rives A (2021) MSA Transformer. In: Proceedings of the 38th International Conference on Machine Learning. pp. 8844–8856

[bRao2014] (2014). Protein-protein interaction detection: methods and analysis. Int J Proteomics.

[bRives2021] (2021). Biological structure and function emerge from scaling unsupervised learning to 250 million protein sequences. Proc Natl Acad Sci USA.

[bRodriques2019] (2019). Slide-seq: a scalable technology for measuring genome-wide expression at high spatial resolution. Science.

[bRual2005] (2005). Towards a proteome-scale map of the human protein–protein interaction network. Nature.

[bSenior2020] (2020). Improved protein structure prediction using potentials from deep learning. Nature.

[bShah2018] (2018). Dynamics and spatial genomics of the nascent transcriptome by intron seqFISH. Cell.

[bSingh2022] (2022). Topsy-Turvy: integrating a global view into sequence-based PPI prediction. Bioinformatics.

[bSledzieski2021] (2021). D-SCRIPT translates genome to phenome with sequence-based, structure-aware, genome-scale predictions of protein-protein interactions. Cell Systems.

[bSong2021] (2021). DSTG: deconvoluting spatial transcriptomics data through graph-based artificial intelligence. BriefBioinform.

[bStickels2021] (2021). Highly sensitive spatial transcriptomics at near-cellular resolution with Slide-seqV2. Nat Biotechnol.

[bTang2023] (2023). SiGra: single-cell spatial elucidation through an image-augmented graph transformer. Nat Commun.

[bThe2019] (2019). RNAcentral: a hub of information for non-coding RNA sequences. Nucleic Acids Res.

[bTheodoris2023] (2023). Transfer learning enables predictions in network biology. Nature.

[bTiwari2021] (2021). Dehydrin in the past four decades: from chaperones to transcription co-regulators in regulating abiotic stress response. Curr Res Biotechnol.

[bUmu2017] (2017). A comprehensive benchmark of RNA–RNA interaction prediction tools for all domains of life. Bioinformatics.

[bVaswani2017] Vaswani A, Shazeer N, Parmar N, Uszkoreit J, Jones L, Gomez AN, Kaiser Ł, Polosukhin I (2017) Attention is all you need. In: Proceedings of the 31st International Conference on Neural Information Processing Systems. pp. 6000–6010

[bVerkuil2022] Verkuil R Kabeli O, Du Y, Wicky BIM, Milles LF, Dauparas J, Baker D, Ovchinnikov S, Sercu T, Rives A (2022) Language models generalize beyond natural proteins. bioRxiv. https://doi.org/10.1101/2022.12.21.521521

[bVickovic2019] (2019). High-definition spatial transcriptomics for *in situ* tissue profiling. Nat Methods.

[bWang2023a] (2023a). Spatial-MGCN: a novel multi-view graph convolutional network for identifying spatial domains with attention mechanism. Brief Bioinforms.

[bWang2023b] (2023b). Construction of a 3D whole organism spatial atlas by joint modelling of multiple slices with deep neural networks. Nat Mach Intell.

[bWang2023c] (2023c). Inferring gene regulatory network from single-cell transcriptomes with graph autoencoder model. PLoS Genet.

[bWang2011] (2011). Molecular mechanisms of long noncoding RNAs. Mol Cell.

[bWang2017] (2017). Accurate *de novo* prediction of protein contact map by ultra-deep learning model. PLoS Comput Biol.

[bWang2023d] (2023d). trRosettaRNA: automated prediction of RNA 3D structure with transformer network. Nat Commun.

[bWang2022] (2022). Single-sequence protein structure prediction using supervised transformer protein language models. Nat Comput Sci.

[bWang2023e] Wang X, Gu R, Chen Z, Li Y, Ji X, Ke G, Wen H (2023e) UNI-RNA: universal pre-trained models revolutionize RNA research. bioRxiv. https://doi.org/10.1101/2023.07.11.548588

[bWang2018] (2018). Three-dimensional intact-tissue sequencing of single-cell transcriptional states. Science.

[bWang2021] (2021). Direct comparative analyses of 10X Genomics Chromium and Smart-seq2. Genomics, Proteomics Bioinformatics.

[bWen2023] Wen H, Tang W, Dai X, Ding J, Jin W, Xie Y, Tang J (2023) CellPLM: pre-training of cell language model beyond single cells. bioRxiv. https://doi.org/10.1101/2023.10.03.560734

[bWu2022] Wu R, Ding F, Wang R, Shen R, Zhang X, Luo S, Su C, Wu Z, Xie Q, Berger B, Ma J, Peng J (2022) High-resolution de novo structure prediction from primary sequence. bioRxiv. https://doi.org/10.1101/2022.07.21.500999

[bWu2019] Wu Z, Pan S, Chen F, Long G, Zhang C, Yu PS (2019) A comprehensive survey on graph neural networks. arXiv. https://doi.org/10.48550/arXiv.1901.00596

[bXu2019] (2019). Distance-based protein folding powered by deep learning. Proc Natl Acad Sci USA.

[bYang2022] (2022). scBERT as a large-scale pretrained deep language model for cell type annotation of single-cell RNA-seq data. Nat Mach Intell.

[bYang2020] (2020). Improved protein structure prediction using predicted interresidue orientations. Proc Natl Acad Sci USA.

[bYe2022] (2022). Single-cell and spatial transcriptomics reveal the fibrosis-related immune landscape of biliary atresia. Clin Transl Med.

[bZeira2022] (2022). Alignment and integration of spatial transcriptomics data. Nat Methods.

[bZhang2021] (2021). Spatially resolved cell atlas of the mouse primary motor cortex by MERFISH. Nature.

[bZhang2023] (2023). Multiple sequence alignment-based RNA language model and its application to structural inference. Nucleic Acids Res.

[bZheng2023] (2023). Deciphering intercellular signaling complexes by interaction-guided chemical proteomics. Nat Communs.

[bZhou2023a] (2023a). Integrating spatial transcriptomics data across different conditions, technologies and developmental stages. Nat Comput Sci.

[bZhou2023b] Zhou Z, Ji Y, Li W, Dutta P, Davuluri R, Liu H (2023b) DNABERT-2: efficient foundation model and benchmark for multi-species genome. arXiv. https://doi.org/10.48550/arXiv.2306.15006

[bZhu2022] (2022). Delineating the dynamic evolution from preneoplasia to invasive lung adenocarcinoma by integrating single-cell rna sequencing and spatial transcriptomics. Exp Mol Med.

[bZuo2022] (2022). Elucidating tumor heterogeneity from spatially resolved transcriptomics data by multi-view graph collaborative learning. Nat Commun.

